# Postnatal dependency as the foundation of social learning in humans

**DOI:** 10.1098/rspb.2024.2818

**Published:** 2025-04-16

**Authors:** Christian Kliesch

**Affiliations:** ^1^ Developmental Psychology, University of Potsdam, Potsdam, Germany

**Keywords:** ontogeny, affordances, social signals, ecological psychology, evolution, embodiment, motor development, agency

## Abstract

Humans have developed a sophisticated system of cultural transmission that allows for complex, non-genetically specified behaviours to be passed on from one generation to the next. This system relies on understanding others as social and communicative partners. Some theoretical accounts argue for the existence of domain-specific cognitive adaptations that prioritize social information, while others suggest that social learning is itself a product of cumulative cultural evolution based on domain-general learning mechanisms. The current paper explores the contribution of humans’ unique ontogenetic environment to the emergence of social learning in infancy. It suggests that the prolonged period of post-natal dependency experienced by human infants contributes to the development of social learning. Because of motor limitations, infants learn to interact with and act through caregivers, establishing social learning abilities and skills that continue to develop as children become less dependent. According to this perspective, at least some key aspects of social development can be attributed to a developmental trajectory guided by infants’ early motor development that radically alters how they experience the world.

## 1. Introduction

Humans are inherently social. Our lives are intertwined with others from birth. We inhabit complex social worlds, built on knowledge passed on and refined over generations. This cumulative social learning is a defining aspect of the human phenotype, and different theoretical approaches have tried to explain its developmental and evolutionary origins. Some theories focus on the existence of domain-specific cognitive adaptations, while others suggest that social learning is itself a product of cumulative cultural evolution based on domain-general learning mechanisms. Despite their disagreements, these theories provide cognitive explanations for the origins of social learning in infancy.

The current paper explores the contribution of humans’ unique ontogenetic environment to the emergence of social learning in infancy. This *Postnatal Dependency Hypothesis* starts with the assumption that species-specific differences emerge out of the interaction between the developing organism and its environment, and the changing opportunities for action that emerge from this interaction [1,2]. In humans, cognitively advanced infants undergo an extensive period of restricted mobility and dependency on caregivers. Subsequently, human infants learn to explore the world *through* others over exploring the world on their own. This establishes a predisposition for social learning even after their motor abilities have caught up. Once established, social learning allows children to acquire increasingly complex social skills, such as linguistic communication and theory of mind, in line with existing theoretical accounts. According to this mechanistic explanation, many uniquely human social learning abilities emerge from humans’ unique ontogenetic environment.

To develop this argument, I contrast current cognitive theories of social learning and introduce embodied and ecological perspectives. Then I compare the ontogenetic trajectories between humans and other species, in particular higher apes. Human ontogeny requires infants to explore the world through others over exploring it on their own. This allows them to learn about social signals and others’ actions in a process similar to sensori-motor exploration. I argue that by acting through others, infants learn to integrate others’ perspectives and affordances and explore how this relates to other important human abilities, such as altercentric cognition, theory of mind and language. I revisit the differences between humans and apes to illustrate how these different ontogenies influence learning. I conclude by contextualizing existing theories from the perspective provided by human post-natal dependency.

## 2. Theories of social learning

Human cultural evolution is rooted in a complex system of social learning. Humans not only imitate others' actions to attain specific outcomes but also copy actions that are opaque or potentially unnecessary for their outcome. This has prompted some theoretical accounts to ground human social learning abilities in uniquely human forms of understanding communicative intentions to identify and prioritize relevant information. These theories can be categorized based on their assumption of whether specific cognitive abilities are derived from abilities already present in apes or other animals, emphasizing its continuity, or represent novel cognitive skills that are unique to humans, emphasizing its discontinuity (cf. [3]).

Domain-specific theories, such as Relevance Theory [4], suggest that understanding communicative intentions is based on unique cognitive adaptation that provides humans with the ability to represent others’ beliefs. This allows humans to search for meaning in others’ communicative actions and interpret them flexibly based on the communicative intent of the speaker. Because the inferential processes required to understand communicative intentions are cognitively complex, Natural Pedagogy [5,6] proposes that infants prioritize information presented in the presence of social signals, such as direct gaze, infant-directed speech and contingent reactivity [7]. This sensitivity is an innate, domain-specific cognitive adaptation that provides the foundation for the transmission of culturally relevant knowledge and behaviours, emphasizing the evolutionary *discontinuity* of social learning in humans.

Other theories also argue that understanding communicative intentions is an essential precondition for social learning in humans. For example, Tomasello [8,9] argues that social learning is based on the ability to engage in joint attention that originates in a domain-general, uniquely human need for cooperation. Similar proposals on the socio-pragmatic origins of social learning have even questioned this discontinuity and argue that even non-human apes have a basic understanding of communicative intentions [10]. Instead, differences in allocating attention provide humans with more advanced forms of understanding communicative intentions later in ontogeny, without requiring domain-specific adaptations [11], thereby suggesting evolutionary continuity between humans and apes.

Heyes [12] rejects domain-specific adaptations for social learning and commits to the continuity between humans and other species. Heyes argues that social learning uses the same domain-general learning mechanisms shared with other species. Uniquely human aspects of social learning, including theory of mind and language, are themselves the result of cumulative cultural evolution. Heyes suggests that the predominance of social learning in humans was caused by quantitative shifts in other cognitive domains, such as increased social tolerance, attention or general learning abilities, leading to a virtuous feedback loop towards increasingly sophisticated learning strategies.

Discontinuity accounts, such as Relevance Theory and Natural Pedagogy, have been criticized because they require sophisticated mindreading skills already at infancy. Even if children can easily identify learning contexts, they still require inferential reasoning to determine *what* to learn [10,13]. Furthermore, other ape species show evidence of understanding basic communicative intentions, making it difficult to specify what a cognitive adaptation contributes [10]. Continuity theories, meanwhile, cannot explain why social learning developed in humans, but not in other animals [3]. I propose that in order to solve this conflict and explain the emergence of uniquely human social learning abilities, we need to consider humans’ unique ontogenetic trajectory as an important source of divergence between humans and other species.

## 3. Grounding social learning in development

Developmental systems theories have highlighted the complex interactions between motor, perceptual and social development and the environment in phenotypical expressions of behaviour [1,2]. Development is an epigenetic process with probabilistic properties. In line with work on probabilistic epigenesis [2], the timing and order of developmental processes become important ontogenetic and evolutionary mechanisms for acquiring phenotypical behaviour. Although perceptual systems across a wide range of species develop in the same order—tactile, vestibular, chemical, auditory and then visual—different developmental outcomes are provided by ontogenetic processes that constrain or enable access to sensory information (e.g. by ensuring that eyes are shut for a period after birth). For example, providing bobwhite quails with visual stimulation in the egg accelerates visual development at the expense of auditory development and prevents chicks from developing a preference for their maternal call by the time of hatching [14]. Similarly, feedback between sensorimotor processes allows mallard ducks to acquire sensitivity to their maternal calls by hearing their own vocalizations in the egg [15]. Although this work has provided important insights into human development [2], there is no comprehensive account that investigates how these interactions affect human socio-cognitive development.

Grounding perceptual and cognitive development in body and environment has been at the centre of Ecological Psychology and Enactivism. According to Ecological Psychology [16,17], perception can only be understood through the relationship between an organism and their environment and the opportunities for action—‘affordances’—it provides. Enactivism conceives organisms as self-organizing systems that bring forth their own existence by interacting with their environment [18]. Cognition is not a process confined to the brain but expressed in all activities, whether mental or physical. Enactivism and Ecological sychology emphasize the link between action and perception in cognition and the role of the organism as an active participant and sense-maker of their specific environment. Whereas Ecological Psychology emphasizes the ontological aspect of the animal–environment interaction, Enactivism explores this relationship from the individual organism making sense of the world they inhabit [19]. These perspectives have provided comprehensive accounts of social cognition and intersubjectivity (e.g. [20,21]), but the specific mechanisms that lead to uniquely human forms of social learning remain unclear.

## 4. Uniquely human altriciality

Animals’ postnatal development can be classified on a spectrum of *precocial* and *altricial* strategies. Precocial animals are born with well-developed perceptual abilities and are able to move soon after birth. Altricial animals are often born blind, bound to a nest and are not able to explore their environment independently, making them dependent on caregivers [22]. Humans and other apes follow a mixed strategy, in which precocial features, such as perceptually and cognitively advanced neonates, are combined with altricial features, such as substantially delayed motor development [23,24]. Although the length of gestation is comparable across apes (chimpanzees: 32 weeks; humans, orangutans and gorillas: 38 weeks [25]), the entire human life history is delayed, and many important markers of development take twice as long [22]. This protracted development is embedded in a long period of post-natal care by parents and alloparents [23,26,27]. This unique developmental trajectory has also been called *secondary altriciality* [23,24].

Despite showing the highest postnatal brain growth within placental mammals [28], human infants are precocial on many cognitive measures. Their brain mass at birth is 26−30% of the adult brain, compared with 40% in chimpanzees [29], but the infant brain shows functionally mature integration of multi-modal information [30,31]. Learning of socially relevant information, such as aspects of maternal language, already takes place before birth [32]. The primary cognitive marker of an altricial development is the slower myelination of axons of nerve cells [28], which slows down information processing [30,31]. This potentially structures perceptual information from the bottom-up and allows post-natal social and environmental experiences to have greater impact on brain development [25,31,33].

Human altriciality is primarily manifested in a protracted motor development, where a comparable maturity to other mammals is reached about 1 year after birth [25]. Differences in motor abilities are also evident compared with other apes. One month after birth, chimpanzees are already more advanced than humans motorically [34]. In the time it takes chimpanzees to walk, human infants are only able to lift their head without support. Chimpanzees learn to walk between 3 and 5 months, while it takes humans 8 to 12 months [35,36]. Even though both species share a comparatively long childhood [22], differences in the onset of locomotion and other motor abilities fundamentally affect how both species engage with their environment during their first year of life [37].

Delays in motor development can be attributed to differences in bodily rather than neural development. Newborn humans show stepping behaviours when held above the ground. Two-month-old babies kick their legs when supine, producing movements similar to a stepping motion. These movements disappear around 3 months because their legs become too heavy. However, infants resume stepping movements if their legs are placed in water [38,39]. These findings suggest that humans are born with ‘a precocial brain, although somewhat disguised by an unwieldy body’ [40].

Different arguments have been made for humans’ extended altriciality. Some authors have argued that, because infants’ head circumference at birth is constrained by the size of the pelvis, development has to continue postnatally, either because of bipedalism [41] or general obstetric constraints in the ape lineages [42]. Other explanations include energy expenditure during pregnancy [22] and selection towards general intelligence [43]. Some authors have also argued that secondary altriciality paves the way to a uniquely human (social) cognition with its own selective benefits [23,24,26,27]. These explanations are not mutually exclusive. Obstetric constraints may have led to infants being born premature, requiring more sophisticated forms of child care and increasing selection for larger brains. Therefore, human intelligence could only develop together with altriciality and post-natal care [43].

## 5. Post-natal dependency as a curriculum for learning

By shifting the onset of motor exploration, human *secondary altriciality* is characterized by a long period of post-natal dependency. This unique environment provides a ‘curriculum for learning’ [44] that ensures that infants learn about social signals as opportunities for interacting with caregivers and their environment before they can engage with the world directly. This perspective highlights the importance of understanding behaviour from the perspective of the organism within their environment, and the opportunities for action it provides.

Already at the time of birth, infants have perceptual preferences for socially relevant stimuli, but these are also shared by other animal species, such as chimpanzees. Preference for face-like stimuli can even be found pre-natally [45]. The preference for faces remains stable even in the absence of exposure to faces during the first months of life [46]. Whereas some accounts attribute these face preferences to domain-specific adaptations [47], others suggest that it is a general feature of binocular development [48]. Human and chimpanzee infants also prefer looking at faces indicating direct, rather than averted gaze [49,50]. Similar preferences exist in the auditory domain: human infants acquire structural aspects of their maternal language *in utero* [32] and after birth, prefer infant-directed speech, which shares many perceptual similarities with speech preferred by other animals [51].

However, there are differences in apes’ and humans’ post-natal development. Young apes spend most of their time being carried by their mothers, holding on to their mother’s fur and taking her perspective. Human infants, however, are either carried or placed on surfaces, prioritizing face-to-face contact [26,52]. The high prevalence of faces in humans is evident in observational data. After birth, infants can focus on objects 12−18 inches from their face, corresponding to the distance of their mother’s face during breastfeeding [25]. During their first months after birth, infants in Western societies predominantly see ceilings and caregivers’ faces [53]. At 2 months, faces are visible for 15 min of each waking hour, declining to 5 min by 12 months [54]. When crawling and walking change infants’ visual experience, children focus more on caregivers’ hands [55,56]. The exposure to faces during these early months contributes to the development of a schematic face model. Children with congenital cataracts that are not treated within 4 months show impaired face processing later in life [44], emphasizing the importance of order in development. As we will see in the next sections, the prevalence of faces and speech in infants’ early environment goes beyond mere exposure but indicates situations in which infants’ ability to interact with their environment is extended by caregivers.

From early on, infants are synchronized with their caregivers on neural and physiological measures that lead to synchronization of affective, behavioural and cognitive states [57,58]. Caregiver–infant synchrony is common across many social animal species during physical contact, but humans are potentially unique in their synchronization in distal interactions [57]. Synchrony during social interactions supports learning [58] and establishing relationships between self and others [59]. This has prompted some authors to suggest that infants’ minimal self extends into their caregivers, and infants only separate self and others later in development [60].

Rather than passively taking in information, infants are intrinsically motivated to learn and explore their environment. Curiosity-driven learning provides children with the motivation to explore the affordances provided by their environment and select potential actions from an open-ended world based on their current knowledge state [61–64]. Infants’ exploratory learning is evident in their attempts to exploit contingencies around them. Exploring sensorimotor contingencies between own and external events allows infants to learn about their influence on the environment [65,66]. Already by 2 months, they learn to kick their foot to engage a mobile above the crib [67]. Direct object manipulation only starts between 3 and 4 months and is accompanied by long periods of passive engagement and onlooking [68]. Only by 5 months do infants learn the prone-to-supine movement [69], freeing up their hands to explore objects. Infants still need to learn to coordinate vision and grasping before they can engage in visually guided reaching [70].

Infants’ behaviours evoke many responses from caregivers. By 2−4 months, infants become sensitive to others’ attention towards them, showing signs of passive joint engagement [68,71]. Rather than exploring objects on their own, they engage with objects through others [68,72]. By 6 months, infants direct a significant amount of their visual attention to social partners [73] and begin to decouple their visual and haptic exploration from caregivers’ actions between 6 and 9 months, before they learn to shift attention between objects held by themselves and their caregivers [72]. Caregivers adjust their behaviour to infants, leading to a loop between infants’ and caregivers’ contingent responses [74,75].

Ostensive signals, such as direct gaze and infant-directed speech, are embedded in caregiver–child interactions and indicate increased opportunities to influence the environment. As caregivers move their heads, attention shifts to hands [76], but hands also predict gaze shifts [77] providing valuable information to learn gaze following [78–80]. These communicative signals not only help to track actions in space but potentially also help to segment actions in time [81].

Because human infants are limited in their ability to act, they need to find other ways of engaging with their surroundings. For example, a child’s unsuccessful reach is reinterpreted when caregivers provide the object to the child [82,83], providing a route towards acquiring pointing. According to Leavens, ‘pointing develops as a solution to a particular kind of developmental problem, characterized by (i) a developing capacity for tool use, (ii) barriers to direct action, and (iii) a history of caregiver responsiveness’ [84]. Crucially, pointing eventually includes informative functions by sharing attention with caregivers rather than just means to acquire objects.

Similar developments take place in the auditory domain. Initially, infants may attend to infant-directed speech due to perceptual features, such as its emotional valence and variability [51,85]. However, infants then discover that it is also highly predictive of everyday social interactions and allows them to coordinate with others [86,87]. Although auditory stimuli can reach learners over longer distances, the auditory system provides a means of establishing the directionality of incoming sounds and linking it to caregivers' actions. As with faces, speech becomes relevant to children not by mere exposure but because it provides infants with opportunities to engage with the environment in meaningful ways [88].

New opportunities for acting on the environment make children susceptible to new environmental distractions and decrease attention to social signals. Reaching children show less attention to social signals compared with non-reachers [89]. Crawling and walking also reduce exposure to social signals. While crawling infants are physically restrained from experiencing others’ faces because they struggle to lift their heads, walking infants rarely lift their heads to look out for others, ‘because they are too busy playing with toys and running around the room’ [35]. Once walking, toddlers are more likely to seek out distal objects [90,91] and spend less time near caregivers [92]. This effect is moderated by the environment: toddlers spend less time near their caregivers in a room full of toys, compared with an empty room [93]. However, walking toddlers now seek out interactions with caregivers to share attention with objects in the environment, boosting word learning [38,90,94]. Caregivers adjust their behaviours to children’s growing skills and provide more learning opportunities to walkers compared with non-walkers [95]. Human infants are immersed in social signals and learn to explore through others, before they are able to explore independently, continuing to prioritize social learning over their own exploration.

Despite systematic cultural differences, parental practices differ primarily in the amount and expression of specific practices [96]. Even if caregivers provide less direct interaction, infants are still embedded in many daily interactions, such as being fed, carried and cleaned, that are highly predictive and relevant for their learning [96], and this leads to limited differences in children’s use of social signals. Gaze following is prevalent even in cultures that engage less in infant-directed communication [97], but is reduced in sighted children of blind parents, where gaze is less predictive [98]. Across many cultures, infants prefer listening to infant-directed speech, and their interest increases during the first year of life [99]. Cultural differences in parental imitation of children’s movements and children’s autonomy also affect developmental markers of self-concept [100,101]. Motor training can boost walking onset, with some children even skipping crawling, but long periods of cradling can also delay motor abilities [38]. However, cultural practices provide only small boosts, and even early walkers still take at least twice as long to walk compared to chimpanzees.

To summarize, social interactions during infancy are formative in the emergence of social behaviours across species. By having a substantially longer period of parental care *before they can engage with the world*, the effect of this dependency is significantly increased in humans and can explain the qualitative difference in the relevance of social signals. Furthermore, infants’ limited motor abilities reduce complexity in their environment. Unable to explore their surroundings independently, infants explore the world through opportunities for action provided by others. These opportunities do not rely on explicit teaching, but are inherent in daily interactions between caregivers and infants. This way, human infants acquire the relevance of social signals before they can explore the environment independently and continue to fulfil their social, epistemic and affective needs through others as they gain independence.

## 6. Embodying self and others in interaction

Numerous studies have found that objects within peripersonal space are processed differently than objects outside of it, and that tools can extend this space. For example, holding a tool can make faraway objects appear closer [102,103]. If tools extend peripersonal space, interacting with others may have similar effects. Infants reaching for out-of-reach objects in the presence of caregivers [83] might *perceive* these objects as closer and reachable. Research in adults has found that instructing someone to perform an action with an object elicits a neural signal akin to the readiness potential, a neural marker of volitional action [104]. The fluidity of self–other boundaries is also evident in kindergarten and primary school children, who misattribute others’ actions as their own in collaborative activities [105,106]. These findings suggest a shared neural encoding of doing an action oneself and telling someone to do it. In this way, humans extend their cognitive system not just into the world [107] but also into others [108].

Integrating others not only fulfils imperative functions but also epistemic needs, such as learning the names of objects, acquiring new objects to explore or being lifted up to see something out of sight. In these interactions, caregivers extend the zone of proximal development [82] by providing children with knowledge otherwise out of their reach. They provide a similar function to motor behaviours in exploring the environment [16]. These triadic interactions constitute instances of joint attention, where infant and caregiver not only attend to an object at the same time but they also attend *together* and provide the basis for the ‘nine month social revolution’ [8]. Rather than understanding them as individual acts of infant and caregiver, they are better conceptualized as examples of the joint infant–caregiver system orienting towards the object of attention; there is no differentiation ‘with respect to the roles played by the child and his helper; it is a general, syncretic whole’ [82].

### (a) Altercentric cognition

In order to ‘act through others’, infants need to take into account others’ perspectives. Recent accounts suggest that infants are born with an altercentric bias and preferentially take others’ perspectives before they become aware of their own [109,110]. Altercentric biases can be found from infancy into adulthood, but recent studies suggest that they are particularly strong in infants dependent on caregivers, and only subside once children can explore on their own [111]. For example, 8-month-old infants exhibit a bias towards remembering information attended with a partner over conflicting information witnessed on their own, but this bias disappears by 12 months [111,112]. However, if others’ beliefs are salient, altercentric biases can still affect adults’ theory of mind performance [113].

According to Bråten [52], perspective-taking in humans and apes is based on an innate mirror neuron system, but humans and apes differ in the preferential encoding of egocentric and altercentric perspectives. In apes, infants are carried on the mother’s back, and the infant and mother’s perspectives coincide. Because of this, apes’ mirror neuron system is innately wired to preferentially encode their *own* perspective. In humans, upright walking affected carrying practices, and infants now faced their caregivers. Consequently, the human mirror neuron system has adapted to preferentially encode *others’* perspectives at birth.

The Postnatal Dependency Hypothesis, however, suggests that altercentrism emerges from sensorimotor learning during early social interactions when their caregivers’ perspective is more relevant for predicting than their own. Infants keep track of what others have and have not seen because they are entangled in close interactions and learn to coordinate their actions through others. However, in interactions in which their own perspective is more relevant (e.g. because the object is under their control), others’ perspectives are ignored. In line with the findings discussed above, altercentric biases are strongest when infants are dependent on caregivers, but subsume later in life, unless specific tasks prime others’ perspectives. Furthermore, altercentric biases are potentially susceptible to differences in interactions, parental styles or cultural differences.

In this view, mirror neurons are not a causal factor of behaviour, but a product of learning processes that link infants’ actions with caregivers’ contingent responses [59]. Research in adults has shown that mirror neuron responses can be trained to mirrored and counter-mirrored finger movements within 1 day [114], providing evidence of a flexible learning mechanism underlying mirror neuron responses. Research in 4-month-old infants further corroborates this interpretation, since mimicry of manual actions depends on infants’ attention to their own hands, but facial mimicry depends on parental imitation of facial gestures [115]. Such a system can flexibly link own and others’ behaviours, provided infants’ actions are reliably matched by others.

Grounding altercentric biases in the species-specific developmental trajectory allows for theoretically more parsimonious explanations of differences between humans and apes that do not require specific adaptations, such as an innate altercentric mirror neuron system. Furthermore, evolutionary selection can act on the adaptive benefits for the overall system of upright walking, higher intelligence and social behaviours, rather than requiring independent selective pressures towards upright walking, intelligence and specific cognitive abilities, such as altercentricism. Humans learn about others’ mental states so extensively because their exploration is entangled with others and their perspectives through cooperative interactions in early childhood [8,116]. This attunement, in turn, originates in their early dependency that ensures others as the primary sources of interaction.

Keeping track of others continues to be important throughout development, providing the basis for human theory of mind. Although the Postnatal Dependency Hypothesis does not make specific predictions on theory of mind development, it suggests that this understanding is initiated by infants’ need to take into account others’ perspectives during early dependency. As children become more mobile, their own perspective becomes increasingly more relevant, providing them with more opportunities to learn when their and other’s perspectives diverge. This ontogenetic trajectory is compatible with suggestions that theory of mind is the product of social learning [12] and early interactions [21,117]. Given their experience of social interactions, other apes might also learn to predict others’ actions in anticipatory looking versions of the false belief task [118,119].

### (b) Language and communication

Language is an important part of human social learning abilities. Establishing linguistic communication and symbolic representations fundamentally restructures cognition, providing access to abstract concepts, scaffolding own actions and communicating mental states [3,12,82,120]. On a societal scale, language allows for the efficient transmission of culturally relevant information, for example, in the form of storytelling and written language.

However, language is also a product of social learning. While the Postnatal Dependency Hypothesis does not make specific predictions about children’s language acquisition, it does provide basic socio-cognitive abilities that are assumed to be necessary for theories of language acquisition. Post-natal dependency embeds children in proto-conversational interactions with caregivers during which their affective, motoric and epistemic actions are embedded and synchronized with others [88,121]. Establishing alignment across multiple levels of interaction is an important feature of human dialogue and enables fast and efficient turn-taking and co-constructing meaning during conversation [122].

In these interactions, ostensive cues, such as direct gaze and infant-directed speech, are embodied signals of attention, allowing infants to engage in communicative interactions without understanding the metarepresentations of communicative intent [123]. Furthermore, post-natal dependency provides infants with many opportunities to practice interacting with others, so that infants can acquire sophisticated forms of pragmatic understanding [8,124].

According to usage-based theories of language acquisition [125,126], children learn linguistic features by using them communicatively with their caregivers. These theories presume an in-principle understanding of communicative interactions, including joint attention, preverbal communication and referential communication. As we have seen in §5, many of these skills can be attributed to a uniquely human ontogeny during the first year of life. By the time children learn to walk, they have acquired the foundations of linguistic communication, such as the referential meaning of words [127], their emerging motor abilities now boost language acquisition by providing novel contexts to use language productively [128].

## 7. Revisiting the comparative perspective

Comparative research on social development suggests that many species exhibit a preference for (human) social signals [49,51], and that early social interactions affect social behaviours later in life. Rhesus macaques show a developmental trajectory of gaze following across lifespan that is similar to humans [129].

Other ape species also have a comparatively long infancy and juvenile period that provides for acquiring social and basic communicative abilities, but their motor development is less constrained [22]. Early social interactions in infancy promote social behaviours later in life. Despite this, humans learn to tune into the more meaningful, predictive aspects of communicative signals beyond what is typically afforded to other animals. Young chimpanzees learn to coordinate with their mothers using gestures learned through ontogenetic ritualization [130] and acquire gestures to coordinate their social activities with other members of their community [131]. These gestures generally initiate dyadic interactions, such as grooming or carrying, rather than coordinating joint activities in triadic contexts. Furthermore, there is little evidence of explicit teaching in apes. Most instances of social learning involve interactions during which adults only tolerate juveniles or involve the learner watching close range, rather than actively support learning [132,133]. There is evidence from orangutans and chimpanzees that adults sometimes support juveniles by providing them with tools [134,135]. However, there are few triadic interactions, where parent and child jointly attend to an object in the environment. While young chimpanzees pay attention to their caregivers to decide what objects to interact with, they rarely look back to their caregivers [136]. Because they can explore the affordances provided by objects independently, they do not need to coordinate with others. This reduces the attention to others’ movements, limiting the integration of others’ actions into the motor system through associative learning (cf. [59]). Consequently, own exploration should favour emulative learning rather than imitative learning.

Apes raised in captivity have shown that novel communicative abilities can emerge from socialization and training [11]. Chimpanzees that grow up with human interactions are more likely to engage in triadic interactions later in life, but these triadic interactions take place after chimpanzees have become mobile [137]. In interactions with humans, bonobos consider their partner’s knowledge when pointing towards hidden objects [138]. Training chimpanzees to imitate human actions strengthens neural connections linked to the mirror neuron system and improves performance on social tasks [139,140]. However, systematic assessments have also identified important differences between humans and apes (e.g. in allocating attention after communicative signals) [11]. For example, captive chimpanzees use human ostensive signals to know when, but not where to search for objects [141].

Laboratory studies provide evidence of the importance of constraining movement to learn novel behaviours. Japanese macaques constrained in a chair can learn to use a rake to reach food. In the absence of other actions, the macaques gradually explored the rake’s affordances and became proficient tool users over repeated sessions [142]. Restricting physical movement enables rhesus monkeys to learn mirror self-recognition, typically not found in this species [143,144]. These findings support the notion that restricting movement allows learners to discover relationships that would otherwise go unnoticed.

Encultured apes, who have grown up in human families, are an extreme example of the influence of caregiving on development. These apes experience similarly reliable caregivers that provide opportunities for actions beyond their immediate reach, thereby supporting the acquisition of informative pointing [84] and rational imitation [145]. Popular reports of chimpanzees growing up in human families [146,147] suggest that they can learn a wide range of social and cultural skills, but fall short of full human cognitive capacities. These largely anecdotal accounts provide evidence of encultured chimpanzees’ abilities, but also report difficulties in constraining them due to their advanced mobility and bodily strength.

The Posnatal Dependency Hypothesis suggests that apes only acquire a human-like understanding of others if they experience similar motor constraints during ontogeny. Such an intervention is ethically not justifiable, but the underlying mechanisms can be studied in simulation studies (cf. [63]). More importantly, differences in motor development affect human and non-human primates within each species’ *typical* development and contribute to the stable formation of species-specific behavioural phenotypes [1].

## 8. Postnatal dependency and theories of social learning

The Postatal Dependency Hypothesis outlines a potential ontogenetic and evolutionary mechanism that grounds human sociality in a species-unique early environment. By linking physiological, cognitive and social development, we do not need to appeal to different selective pressures for each adaptation in human evolution and ontogeny. Instead, the joint emergence of social, cognitive and physiological abilities can be explained by their interdependence and shared causal explanations.

The Postnatal Dependency Hypothesis extends current theories of social learning by providing a mechanistic account of children’s social learning abilities, but also challenges some of their assumptions and conclusions. In line with existing theories, it suggests that social learning is a defining feature of the human phenotype and its foundations emerge in early development [5,8]. However, it challenges nativist accounts that argue for specific cognitive adaptations that process mental states of others (cf. [4]) or for infants to recognize communicative intentions [5]. By illustrating a potential route towards acquiring an understanding of communicative relevance in infancy, it complements non-nativist pragmatic accounts to social learning [8,10] and domain-general learning accounts [12]. By immersing children into an extended period of postnatal care, it provides an embodied route towards recognizing communicative intentions [10]. Grounding social cognition in secondary altriciality and its subsequent dependency can potentially explain why apes can develop foundational socio-pragmatic abilities, but their expression in humans goes beyond what is found in chimpanzees.

The basic mechanisms that shape infants’ learning are compatible with general learning approaches such as curiosity-driven learning [61] and associative sequence learning [59]. However, the resulting behaviours prioritize others as opportunities for action and interaction, prioritizing socially presented information. By providing an embodied account rooted in humans’ extended period of dependency, the Postatal Dependency Hypothesis provides an explanation of how domain-general, non-social learning strategies can lead to the emergence of social biases in learning.

There are a number of aspects that post-natal dependency cannot account for, which I will briefly discuss as follows.

Like other theories of social learning, the Postnatal Dependency Hypothesis focuses on the emergence of socio-communicative skills in early childhood and makes few predictions about the content of socially transmitted information. Although it provides an ontogenetic foundation for how language [126] and theory of mind [12,21,117] could be acquired through social learning, the Postnatal Dependency Hypothesis makes only general predictions about their timing and expression.

The Postnatal Dependency Hypothesis highlights the interaction between environment and organism in the development of species-specific behaviours [1,16]. Innate contributions play an important role in this relationship. However, as suggested here, they do not need to be realized on a cognitive level, but can also be embodied in a unique developmental trajectory that changes how an organism interacts with its environment. This does not rule out that domain-specific cognitive adaptations contribute to human social learning abilities. However, it raises the question of how these adaptations function in the wider context of human ontogeny.

Postnatal dependency is linked to other physiological and cognitive changes during human evolution, such as increased brain capacity, slower myelination, upright walking and hairlessness, which in turn affect how humans interact with their environment. The Postnatal Dependency Hypothesis makes no causal claims about which adaptations were selected for originally. Social learning could be a by-product of an initial selection for bipedalism, or general intelligence was selected for, with bipedalism as the ontogenetic realization. Once established, cumulative cultural evolution can affect organisms’ environmental niche and subsequently lead to additional genetic adaptations that influence the phenotype [12].

## 9. Conclusion

Vygotsky wrote that ‘[t]he path from object to child and from child to object passes through another person’ [82]. According to the Postnatal Dependency Hypothesis, the reason for this detour can be found in infants’ limited motor abilities. From the moment they are born, they are embedded in interactions with others and have to fulfil bodily and epistemic needs through others.

Humans follow a developmental strategy that is radically different from other species. They are born with a highly developed cognitive and perceptual system, but their bodies constrain how they can explore their environment. This has profound effects on their ‘curriculum for learning’. Instead of being able to interact with the world directly, they explore it in interaction with their caregivers. Infants learn to coordinate with others in the same way that they eventually learn to coordinate their own actions. This primes infants towards others’ perspectives and eventually teaches them when their own and others’ perspectives diverge. It is this peculiar environment that provides the foundations of social learning in humans.

## Data Availability

This article has no additional data.
